# Supplementary material for: The discovery of novel noncoding RNAs in 50 bacterial genomes
Source: Nucleic Acids Res. 2024 Apr 22;52(9):5152–65. doi: 10.1093/nar/gkae248 (PMC11109978; doi:10.1093/nar/gkae248)
Supplement: gkae248_Supplemental_Files [file gkae248_supplemental_files.zip › Narunsky 50 Genomes RNA Motifs Files.zip!/Publication/56-D_turgidum/Figures/LRC-56-6-NC_011661_1_IGR_26_299625_299813.sto.pdf]

LRC-56-6-NC_011661_1_IGR_26_299625_299813.sto.r2r

5′- U U U U U Y G C U U G A C A A A A A A U U A U R A R U R A R U A A A A A U U R Y U U A R G C A A A C G U U U A U A U U A G C G U U U G C A A A A U U A A A A R G G G G G
